# Fabrication of healable and flame-retardant polymer films based on phosphonate dynamic covalent crosslinking networks

**DOI:** 10.1039/d6ra08494j

**Published:** 2026-10-21

**Authors:** Kazuho Maeda, Yota Okuno, Yasuhiko Iwasaki

**Affiliations:** a Graduate School of Science and Engineering, Kansai University 3-3-35, Yamate-cho Suita Osaka 564-8680 Japan; b Department of Chemistry and Materials Engineering, Faculty of Chemistry, Materials and Bioengineering, Kansai University 3-3-35 Yamate-cho Suita-shi Osaka 564-8680 Japan yasu.bmt@kansai-u.ac.jp; c ORDIST, Kansai University 3-3-35 Yamate-cho Suita-shi Osaka 564-8680 Japan

## Abstract

Phosphoester linkages are attractive not only for flame retardancy but also dynamic covalent networks (DCNs) while preparing self-healing materials. However, thermal activation of these linkages may induce irreversible structural changes by condensation or volatile product evolution. In this study, phosphonate crosslinkages were investigated as an alternative dynamic linkage and directly compared with phosphoester crosslinkages in polymer networks based on poly(2-hydroxyethyl methacrylate) (PHEMA). Phosphoester- and phosphonate-crosslinked films were prepared using 2-methoxy-2-oxo-1,3,2-dioxaphosphorane or 2-methyl-2-oxo-1,3,2-dioxaphosphorane, respectively. Upon thermal treatment, the phosphoester-based DCN exhibited irreversible changes in mechanical properties and mass loss, resulting from the evolution of volatile byproducts during transesterification. Conversely, the phosphonate-based DCN maintained its mechanical properties and catalyst-free dynamic bond exchangeability with an activation energy of 122.0 kJ mol^−1^, enabling repeatable thermal healing with approximately 99% recovery of the original tensile toughness. The two networks also exhibited distinct flame-retardant behaviors. Although both networks showed self-extinguishing behavior after flame exposure, the phosphonate-based DCN extinguished much more rapidly and exhibited substantially reduced internal thermal damage. Furthermore, the polymer film containing phosphonate-based DCNs exhibited superior flame-retardant properties owing to the instantaneous flame extinction capabilities by the P–C bonds. These results demonstrate that phosphonate-based dynamic crosslinking provides a promising strategy for designing multifunctional polymer networks that combine structural stability, thermal healing intrinsic flame retardancy.

## Abstract 2

To control the transesterification reaction in phosphorus-containing dynamic covalent networks (DCNs), phosphonate-based DCNs ensure stable mechanical and dynamic properties while simultaneously imparting superior intrinsic flame retardancy.

## Graphical abstract

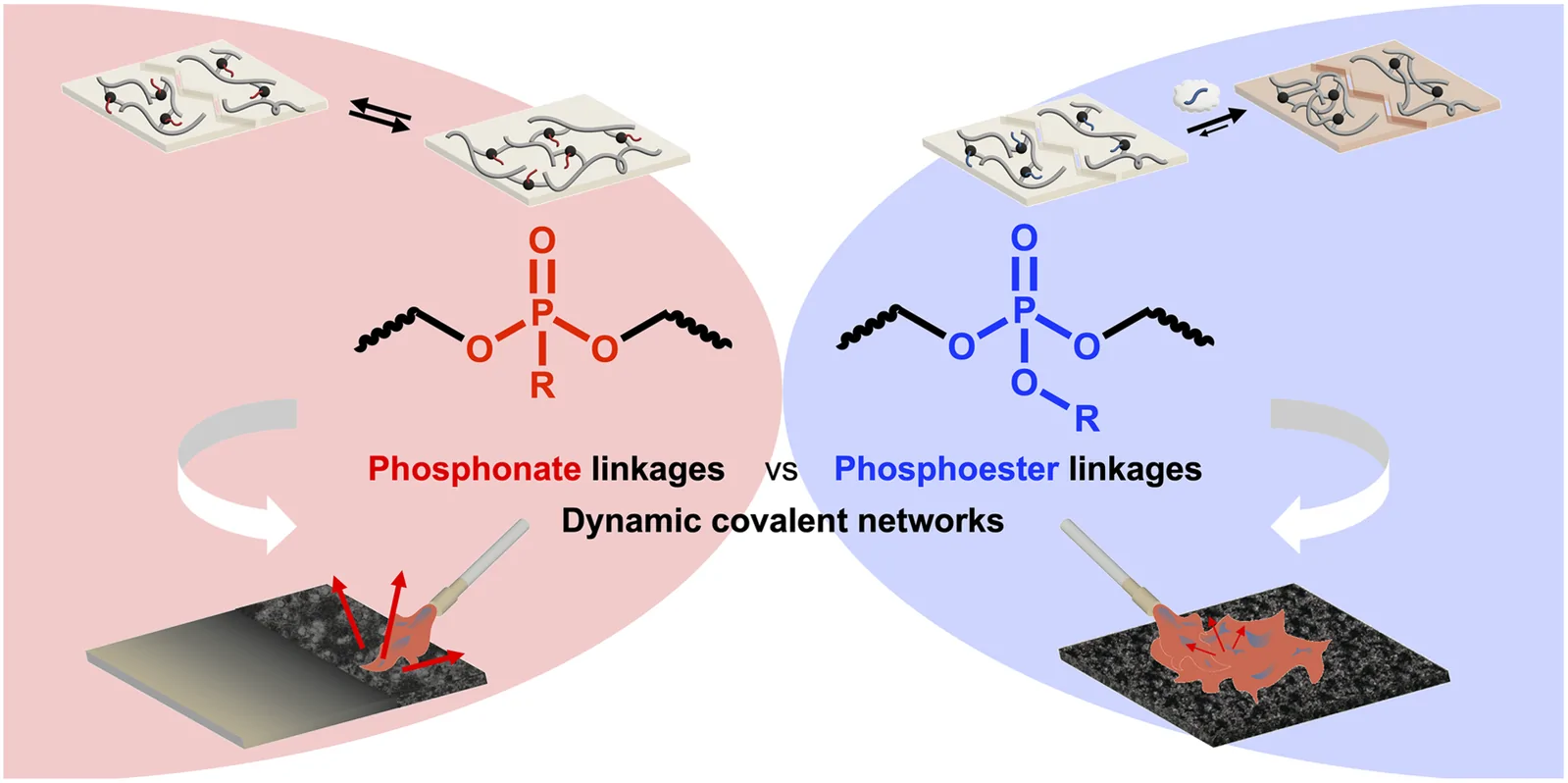

## Introduction

Conventional thermoplastic polymers undergo irreversible property degradation upon recycling.^1,2^ Therefore, dynamic covalent networks (DCNs) are a promising class of recyclable polymeric materials.^3,4^ Among these DCNs, transesterification-based networks are actively studied as they can be easily incorporated into conventional polymer materials.^5–8^ Carboxylate ester-based DCNs are the most readily available common; however, efficient bond exchange typically requires elevated temperatures and substantial catalyst amounts.^9^ These specifications may induce unintended structural changes, compromising material stability.^10^

Recently, phosphoester-based DCNs have emerged as an alternative to conventional carboxylate ester networks because the energy level of the reaction intermediate is lower than that of carboxylate esters, allowing the bond exchange to proceed under milder conditions.^11^ Furthermore, phosphorus presence provides intrinsic flame-retardant properties to the materials.^12–17^ Despite these advantages, phosphoester-based DCNs remain susceptible to structural deterioration under the high temperature conditions required to induce bond exchange. At elevated temperatures, condensation between P–OH bonds^18^ and transesterification accompanied by the loss of alkoxy groups from phosphoester side chains^19^ alter the material such that further bond exchange is severely inhibited.

The flame-retardant performance of phosphoester-based DCNs also presents an inherent limitation. Their primarily function through a condensed phase, where polyphosphoric acid generated during thermal decomposition promotes dehydration and carbonization, resulting in the formation of a protective char layer on the material surface.^20^ However, sufficient time is required for the development of an effective char barrier,^12^ limiting its ability to suppress combustion upon ignition.

To address these limitations, we focused on phosphonate-based DCNs. A phosphonate linkage contains two reversible P–O–C bonds, one irreversible P–C bond, and a P=O bond. This structure suppresses transesterification accompanied by side reactions during network rearrangement at elevated temperatures, maintaining the original network structure and crosslinking density. Furthermore, the P–C bond is more susceptible to cleaving at high temperatures than the P–O bond, owing to its lower bond dissociation energy.^20,21^ This thermal decomposition can readily release volatile phosphorus-containing radicals that instantaneously scavenge the high-energy radicals responsible for combustion chain reactions.^22^ This gas-phase flame-retardant mechanism is expected to instantaneously suppress combustion. Although several phosphonate-based DCNs have been reported,^16,23^ a systematic comparison of the bond-exchange behavior, structural stability, and flame-retardant properties of phosphoester- and phosphonate-based crosslinkages has not yet been explored.

In this study, phosphoester- and phosphonate-based DCNs containing methoxy and methyl groups, respectively, at the crosslinking sites were prepared and systematically compared. Thermally induced transesterification was used to evaluate the bond-exchange behavior of both networks, emphasizing on their structural stability, mechanical properties, and flame-retardant properties. The phosphoester-based DCNs exhibited irreversible structural degradation upon thermal treatment, whereas the phosphonate-based DCNs retained their structural stability and mechanical properties without side reaction during transesterification. In addition, the phosphonate-based DCNs exhibited effective self-extinguishing behavior. To the best of our knowledge, this is the first systematic study directly comparing phosphoester and phosphonate dynamic crosslinking networks within structurally comparable polymer networks.

## Experimental

### Materials

2-Hydroxyethyl methacrylate (HEMA, Fujifilm Wako Pure Chemical Co., Osaka, Japan) was purified by vacuum distillation prior to use. 2,2′-Azobisisobutyronitrile (AIBN, Fujifilm Wako Pure Chemical Co., Osaka, Japan) was purified by recrystallization from ethanol prior to use. 2-Methoxy-2-oxo-1,3,2-dioxaphospholane (MP; Fujifilm Wako Pure Chemical Co., Osaka, Japan) was prepared following established protocols,^24,25^ purified by distillation under reduced pressure, and stored under an Ar atmosphere at 4 °C. Similarly, 2-methyl-1,3,2-dioxaphospholane 2-oxide (MeP; Fujifilm Wako Pure Chemical Co., Osaka, Japan) was synthesized according to the literature,^26^ purified by vacuum distillation, and preserved at −30 °C under Ar atmosphere. Poly(2-hydroxyethyl methacrylate) (PHEMA; Fujifilm Wako Pure Chemical Co., Osaka, Japan) was synthesized *via* conventional free-radical polymerization initiated by AIBN and subsequently purified by reprecipitation in isopropyl ether. The number-average molecular weight (*M*_*n*_) and weight-average molecular weight (*M*_w_) of the synthesized PHEMA were evaluated by gel permeation chromatography (GPC; Shimadzu Co., Kyoto, Japan) utilizing a refractive index detector. The GPC system was equipped with PSS PFG analytical size-exclusion columns (100 and 1000 Å; Agilent, Amherst, MA, USA), with trifluoroethanol as the eluent. Calibration was performed using poly(methyl methacrylate) standards (Tosoh Co., Tokyo, Japan). The synthesized PHEMA had an *M*_*n*_ of 3.6 × 10^5^ g mol^−1^ and *M*_w_/*M*_*n*_ of 2.89.

### Preparation of crosslinked polymer films

Crosslinked polymer films were prepared according to a previously reported procedure.^27^ First, PHEMA was dissolved in *N*,*N*-dimethylformamide (DMF) to obtain a 20 wt% polymer. Either MP or MeP was introduced to the PHEMA solution at the desired concentration, and the mixture was vigorously stirred under reduced pressure at 25 °C for 5 min. The resulting mixture was cast into a polytetrafluoroethylene mold and heated at 100 °C for 2 days to induce transesterification, yielding phosphoester- or phosphonate-crosslinked polymer films, respectively. The cured films were immersed in 2-propanol for 2 days to remove unreacted residual compounds. After washing, the purified networks were recovered and dried under vacuum at 60 °C.

### Determination of phosphorous content in crosslinked polymer films

Colorimetric analysis was employed to quantify the molar ratio of HEMA units to phosphorus-containing crosslinking units (phosphoester or phosphonate units). Film samples (10–15 mg) were completely digested in perchloric acid at 180 °C to afford a homogeneous solution. The digested samples were subsequently mixed with aqueous ammonium molybdate tetrahydrate (1.26 wt%) and ascorbic acid (5.03 wt%), along with the addition of Milli-Q water. The mixtures were homogenized using a Vortex-Genie 2 mixer (Kenis, Ltd, Osaka, Japan) and incubated at 80 °C for 5 min. The resulting solutions were transferred to a 96-well plate, and their absorbance at 820 nm was measured using an SH-9000Lab microplate reader (Corona Electric Co., Ltd, Ibaraki, Japan). Phosphorus concentrations were determined from a calibration curve prepared using standard solutions of disodium hydrogen phosphate dodecahydrate. The molar ratio of HEMA units to phosphorus-containing units was calculated from the measured phosphorus content, assuming one phosphorus atom per phosphoester or phosphonate crosslinking unit. Data are presented as the mean ± standard deviation derived from at least three independent measurements (*n* = 3).

### Evaluation of thermal-healing ability

For the cyclic thermal-healing test, disk-shaped films (diameter: 8 mm; thickness: ∼0.7 mm) were prepared. Each film was cut along its diameter to approximately 7.5 mm using a scalpel, leaving a small uncut region at one end to serve as a joint. The partially cut film was placed in a Teflon mold designed to apply compressive force perpendicular to the cut surfaces, ensuring intimate contact, and heated at 120 °C. This temperature was selected because it falls within the typical healing temperature range (100°C–160 °C) reported for similar phosphoester-based^12,14,28^ and phosphonate-based DCNs,^16,23^ while minimizing potential side reactions involving phosphoester linkages at elevated temperatures.^28^ One healing cycle was defined as the cutting and subsequent thermal-healing process, and the procedure was repeated for cyclic evaluation.

For quantitative evaluation of healing efficiency by tensile testing, larger disk-shaped specimens (diameter: 40 mm; thickness: ∼0.7 mm) were prepared. Each specimen was cut for approximately 20 mm at the center along its diameter, leaving both edges intact. The cut specimens were healed for predetermined periods under the same conditions described above. After healing, the disks were punched into dumbbell-shaped specimens, ensuring that the healed interface was positioned within the gauge length for tensile testing. Following the tests, the tensile toughness (MJ m^−3^) of the specimens was calculated from the area under the stress–strain curve by numerical integration (trapezoidal rule) according to the following equation:*U* = ∫*σ* d*ε*where *σ* is the engineering stress and *ε* is the engineering strain. The healing efficiency was then determined as the ratio of the tensile toughness of the healed specimen to that of the pristine specimen. All measurements were repeated for multiple specimens (*n* = 3) to evaluate the standard deviation, and the detailed values are summarized in Table S1.

### Combustion tests

Combustion behavior was evaluated using rectangular film specimens (5 mm × 20 mm × (0.4 ± 0.02) mm). Each specimen was held with tweezers, and a gas burner flame was applied to one end for 6 s. After removal of the flame, the combustion behavior of the film was observed. Each sample was tested in triplicate.

### Characterization methods

#### Nuclear magnetic resonance spectroscopy

^31^P nuclear magnetic resonance (NMR) spectra were recorded at 30 °C using a JEOL JNM-ECZS spectrometer (400 MHz; JEOL Co., Tokyo, Japan). Dimethyl sulfoxide-*d*_6_ was utilized as the solvent, and tetramethylsilane was used as the internal reference.

#### Fourier transform infrared spectroscopy

Attenuated total reflection Fourier transform infrared (ATR-FTIR) spectra were recorded using an FT/IR-6300 spectrometer (JASCO, Tokyo, Japan). Each spectrum was collected over the range of 4000–500 cm^−1^ with a spectral resolution of 4 cm^−1^ and 256 accumulated scans.

#### Tensile testing

Uniaxial tensile tests were performed using a universal testing machine (AGS-500NX, Shimadzu Co., Kyoto, Japan) at a constant crosshead speed of 5.0 mm min^−1^. Dumbbell-shaped specimens with a gauge length of 12 mm, width of 2 mm, and a thickness of approximately 0.8 mm were used. Data are presented as the mean ± standard deviation derived from at least three independent measurements (*n* = 3). The Young's modulus was determined from the initial linear region (strain of 0.2–0.5%) of the stress–strain curves.

#### Isothermal thermogravimetric analysis

Isothermal thermogravimetric analysis (TGA) was performed using an EXSTAR6200 instrument (Seiko Instruments Inc., Chiba, Japan). Samples (5–10 mg) were placed in aluminum crucibles and analyzed under a N_2_ atmosphere for 3 h at 120 °C.

#### Scanning electron microscopy

The sample surfaces and cross-sections collected after the combustion test were observed using a JCM-7000 scanning electron microscope (SEM; JEOL, Akishima, Japan). SEM measurements were conducted at an accelerating voltage of 15.0 kV and a working distance of 16 mm.

#### Microscale combustion calorimetry

Microscale combustion calorimetry (MCC) was performed in accordance with ASTM D7309 Method A using an MCC-3X-UL instrument (Deatak, Illinois, USA). Samples (approximately 10 mg) were thermally decomposed by heating from 150 to 750 °C at a constant heating rate of 1 K s^−1^. Each sample was tested in triplicate.

## Results and discussion

### Ring-opening reactivity and incorporation efficiency of cyclic phosphorus compounds

Following our previous work,^27^ crosslinked polymer networks containing phosphoester or phosphonate crosslinkages were prepared using PHEMA and the corresponding cyclic phosphorus compounds, MP or MeP, at various feed ratios (Fig. 1). To evaluate the effect of the cyclic monomer feed ratio on the incorporation of phosphorus-containing units into the polymer networks, the molar feed ratio (*X*) of MP or MeP to HEMA repeat units was varied from 0.3 to 3. The incorporated molar ratio (*Y*) of the phosphoester or phosphonate units relative to HEMA units was determined using the molybdenum blue method. The networks prepared using MP and MeP are denoted as PHPO_*Y*_ and PHPC_*Y*_, respectively.

**Fig. 1:**
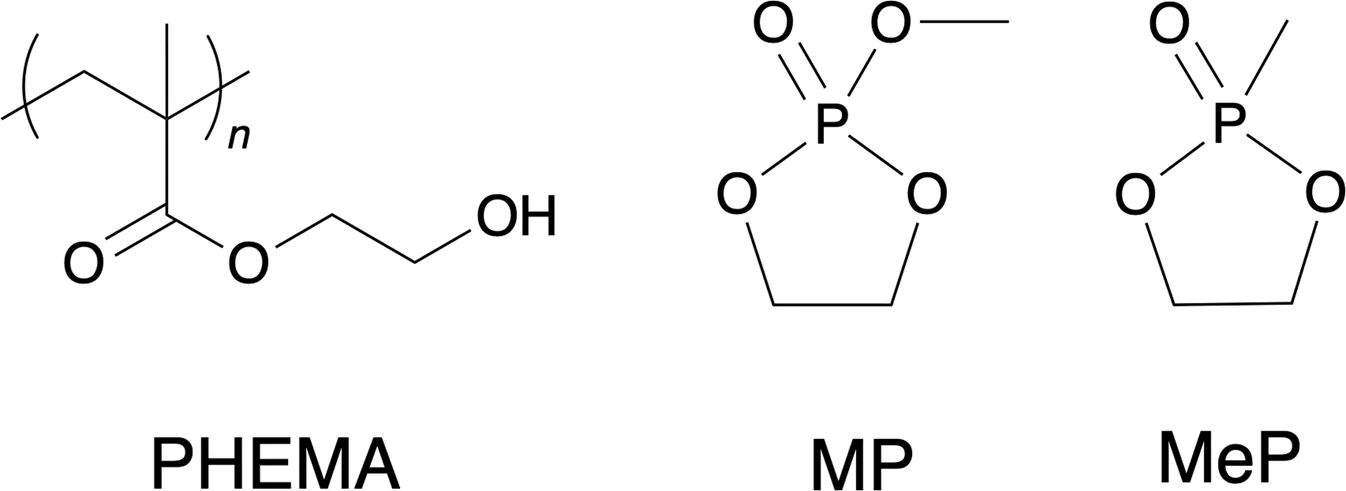
Chemical structures of poly(2-hydroxyethyl methacrylate) (PHEMA), 2-methoxy-2-oxo-1,3,2-dioxaphospholane (MP), and 2-methyl-1,3,2-dioxaphospholane 2-oxide (MeP).

The relationship between *X* and *Y* is shown in Fig. 2. For the PHPO_*Y*_ networks, the incorporation of phosphoester units (PO) increased continuously with MP feed ratio. In contrast, the incorporation of phosphonate units (PC) in the PHPC_*Y*_ networks reached a plateau, with *Y* remaining at only 0.21 despite further increases in the MeP feed ratio. To elucidate the origin of these distinct incorporation behaviors, the progress of the ring-opening reactions of MP and MeP was examined by ^31^P NMR spectroscopy.

**Fig. 2:**
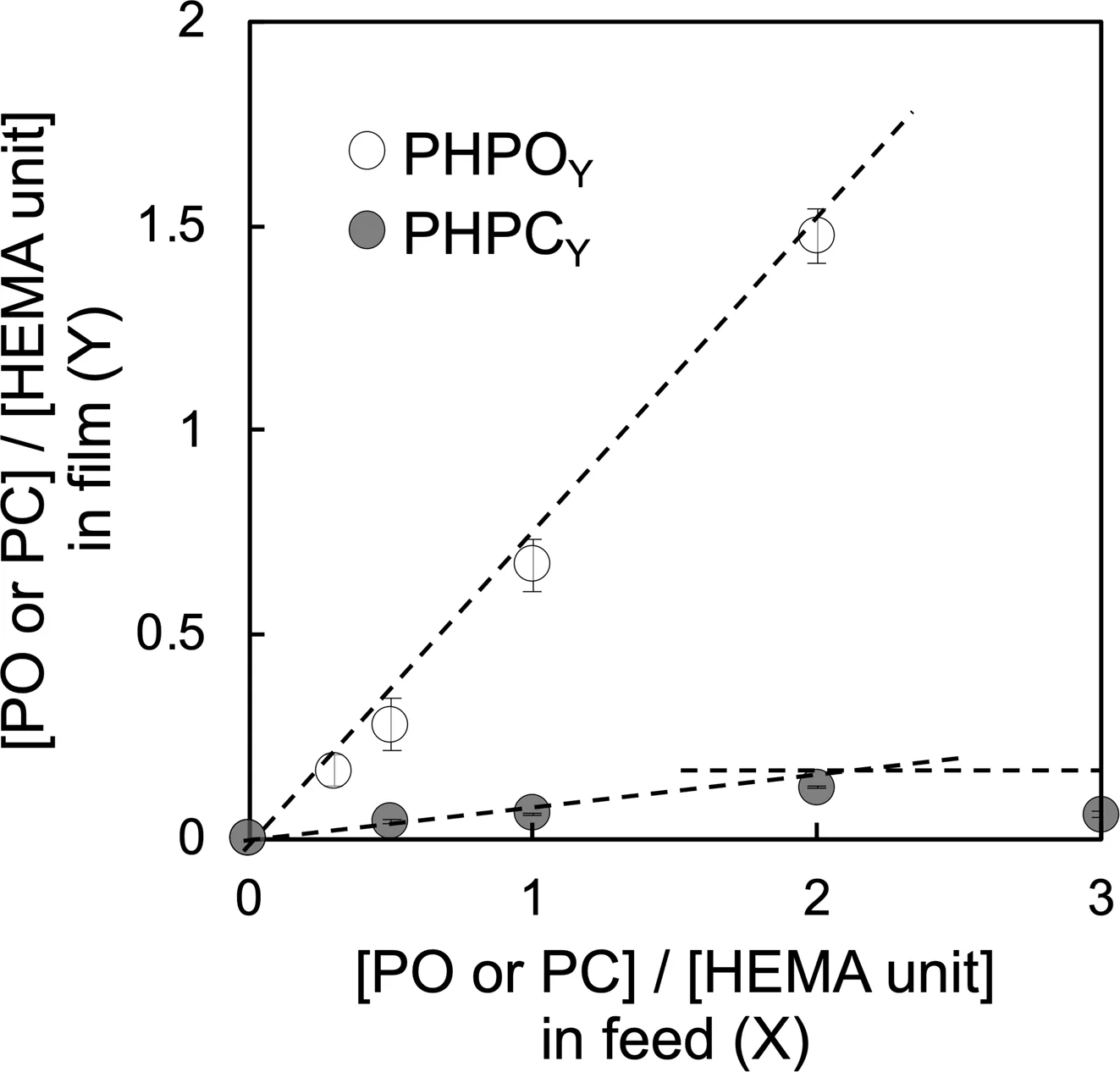
Relationship between the initial feed molar ratio of phosphoester (PO) or phosphonate (PC) units to HEMA units (*X*) and the incorporated molar ratio of PO or PC units to HEMA unit in the resulting films (*Y*). PO and PC are used exclusively in this figure to denote phosphoester and phosphonate units, respectively.

Reaction mixtures containing HEMA units and MP or MeP at a 1 : 1 molar ratio were analyzed before and after heating for 1 h. Samples could not be recovered after longer heating times because the initially homogeneous reaction mixtures became crosslinked networks. The resulting ^31^P NMR spectra are shown in Fig. 3a and b. Before heating, the signals corresponding to the cyclic reactants, MP (18.46 ppm) and MeP (48.75 ppm) accounted for >90% of the total phosphorus-containing species in their respective reaction mixtures. After heating, new signals appeared at 0.58 ppm for the MP-containing reaction mixture and at 28.09 ppm for the MeP-containing reaction mixture. Previous studies have reported that the ^31^P NMR signals of the ring-opened products of MP and MeP occur at higher field than those of the corresponding cyclic monomers.^26,29^ Accordingly, these newly observed signals were assigned to the respective ring-opened species. When the integral of the cyclic monomer signal was normalized to 1.00, the integral of the ring-opened species was 1.57 for the MP-containing reaction mixture but only 0.24 for the MeP-containing reaction mixture. These results indicate that MP undergoes ring-opening substantially more readily than MeP under the present reaction conditions, which accounts for the lower incorporation efficiency of PC units into the PHPC network.

**Fig. 3:**
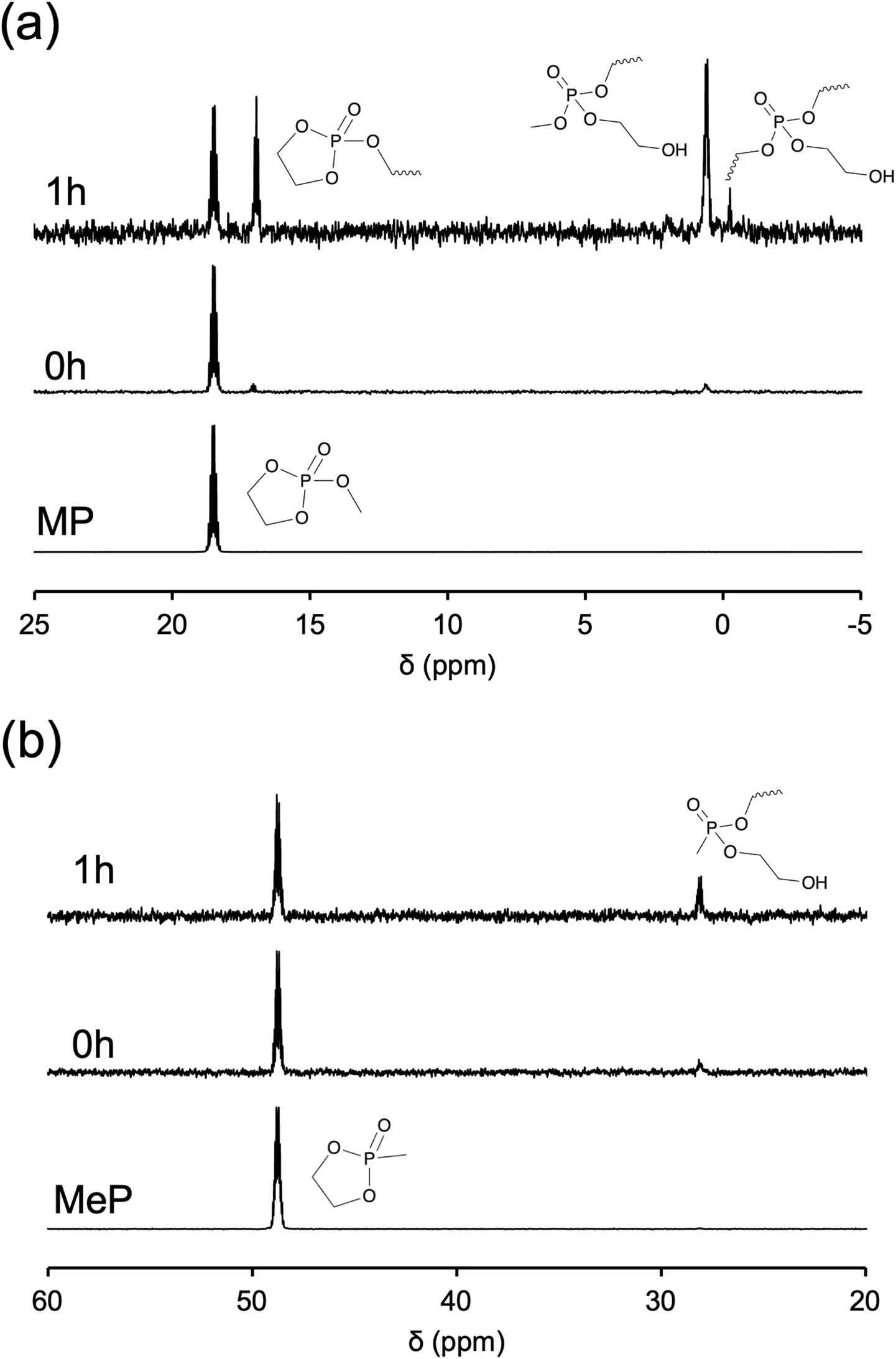
^31^P NMR spectra recorded during the ring-opening reaction of (a) MP and (b) MeP in the reaction mixtures used to prepare the phosphoester- and phosphonate-based DCNs, respectively.

To enable a direct comparison of the effects of phosphoester and phosphonate crosslinking while minimizing differences in phosphorus-unit content, the following experiments were conducted using PHPO_0.21_ and PHPC_0.21_. Both networks contained an identical incorporation ratio of 0.21 phosphorus-containing units relative to HEMA repeat units.

### Characterization of phosphoester- and phosphonate-based DCNs

The formation of crosslinked network structures in PHPO_0.21_ and PHPC_0.21_ was first examined by a film dissolution test in DMF, a solvent in which the constituent components are soluble. As shown in Fig. 4a, PHEMA completely dissolved in DMF, whereas PHPC_0.21_ and PHPO_0.21_ retained their macroscopic shapes, indicating the formation of insoluble crosslinked networks.

**Fig. 4:**
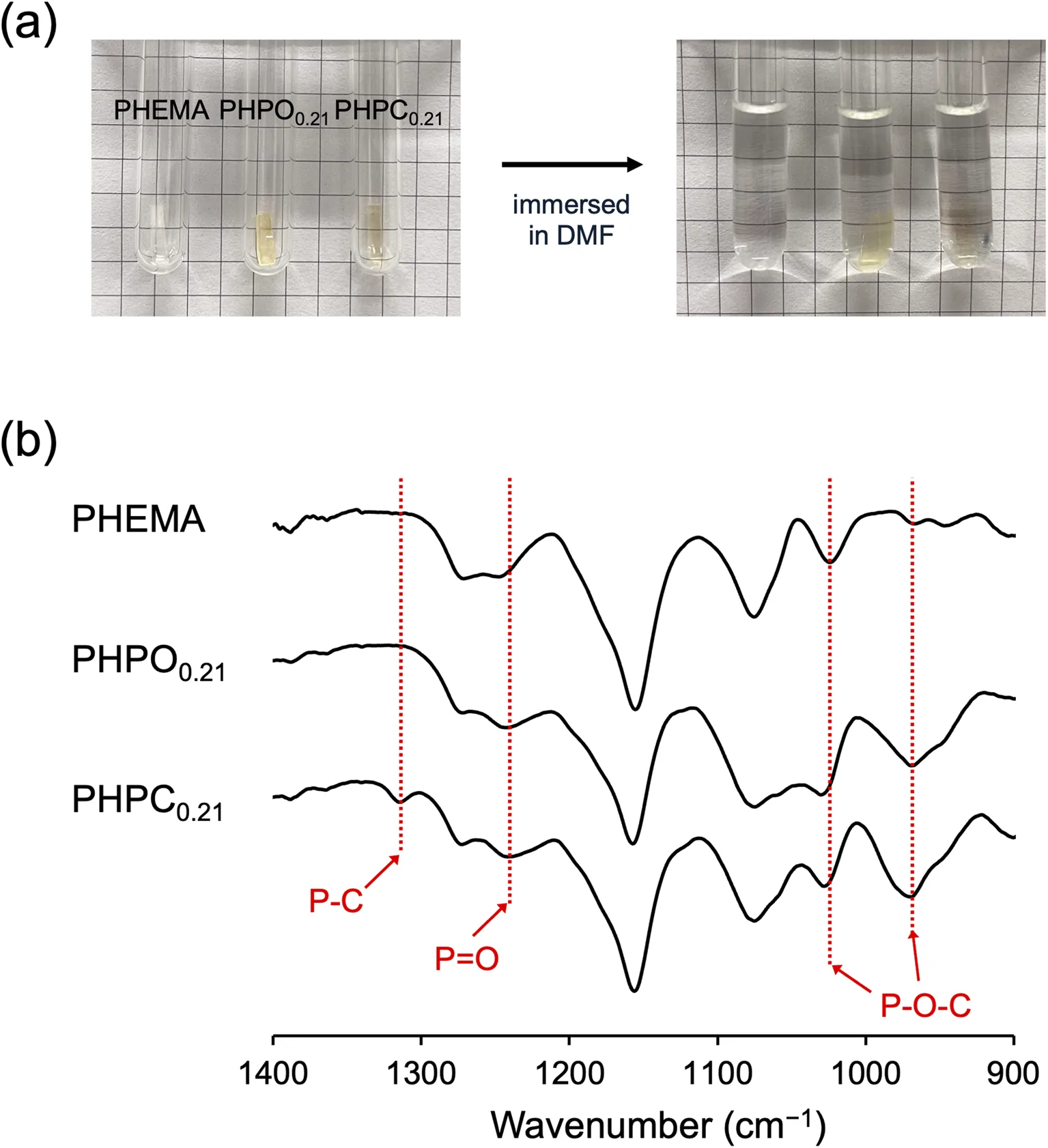
(a) Photographs of PHEMA, PHPO_0.21_, and PHPC_0.21_ before and after immersion in DMF. (b) ATR-FTIR spectra of PHEMA, PHPO_0.21_, and PHPC_0.21_.

The chemical structures of PHEMA, PHPO_0.21_, and PHPC_0.21_ were further characterized by FTIR spectroscopy (Fig. 4b and S1). PHPO_0.21_ and PHPC_0.21_ exhibited absorption bands at approximately 970 and 1025 cm^−1^, assigned to P–O–C stretching vibrations, as well as a band at approximately 1230 cm^−1^ attributable to P=O stretching. Compared with PHEMA, the OH stretching band (3200–3600 cm^−1^) shifted to lower wavenumbers in both networks, suggesting the formation of hydrogen-bonding interactions between the hydroxyl groups of PHEMA and the phosphoryl (P=O) groups introduced at the crosslinking sites. In addition, an absorption band at approximately 1315 cm^−1^, assigned to P–C stretching, was observed exclusively for PHPC_0.21_, consistent with the presence of phosphonate linkages.

These peaks are attributed to phosphoester or phosphonate bonds. Collectively, these results support the successful formation of crosslinked polymer networks containing phosphoester and phosphonate crosslinkages in PHPO_0.21_ and PHPC_0.21_, respectively. The presence of P–O–C linkages further indicates that both networks contain potentially exchangeable bonds capable of participating in transesterification.

### Thermal-healing ability of phosphoester- and phosphonate-based DCNs

A key advantage of DCNs is their ability for healing and reprocessing induced by external stimuli (*e.g.*, thermal treatment or photoirradiation), despite possessing a crosslinked network structure.^6,30,31^ However, for their practical applications as sustainable materials, maintaining their initial mechanical properties without degradation after such thermal processes is a crucial requirement. To evaluate the effect of thermal treatment (at 120 °C) on the mechanical properties, tensile tests were performed on the original films and films subsequently heated at 120 °C for 2 h (Fig. 5). For PHPO_0.21_, the thermal treatment increased the Young's modulus by approximately 1.38 times relative to that of the original film (Fig. 5a). In contrast, PHPC_0.21_ exhibited almost no change in Young's modulus after heating (Fig. 5b).

**Fig. 5:**
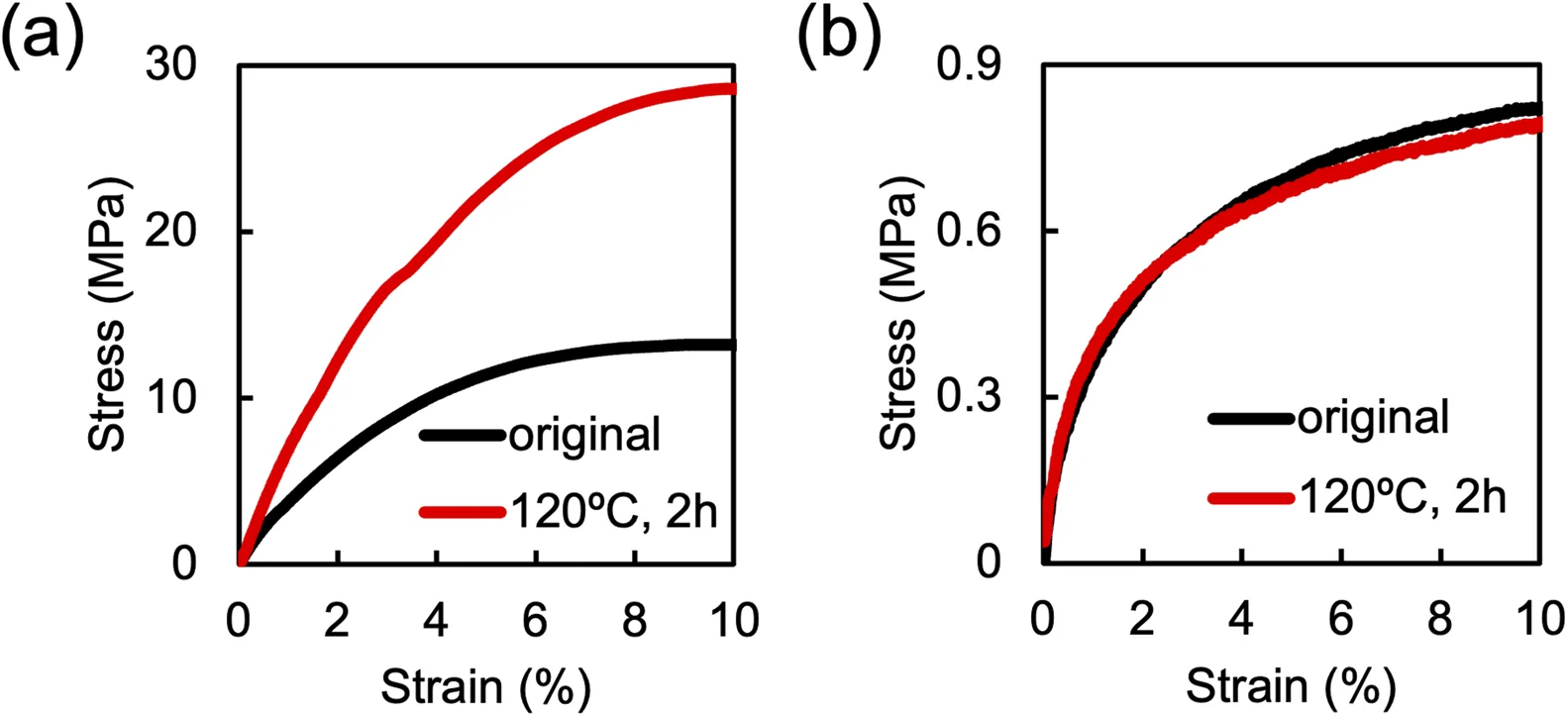
Stress–strain curves of (a) PHPO_0.21_ and (b) PHPC_0.21_ before and after thermal treatment at 120 °C for 2 h. Black and red lines represent the original and heat-treated samples, respectively.

Such a change in mechanical properties is closely related to the thermal stability and subtle chemical structural alterations of the material during heating. To investigate the origin of the mechanical-property change, isothermal TGA measurements were performed at 120 °C (Fig. 6a). After 3 h, PHPO_0.21_ exhibited a weight loss of approximately 1.5%, whereas no detectable weight loss was observed for PHPC_0.21_. We performed FTIR measurements before and after heating to identify the chemical origin of this mass loss; however, direct spectroscopic confirmation was hindered because the characteristic absorption peaks of the suspected eliminated molecules (such as methanol or ethylene glycol) severely overlapped with the broad signals of the bulk polymer network. S. Majumdar *et al.* reported that phosphotriester-based DCNs containing ethoxy group substituents at the crosslinking sites undergo thermal weight loss through elimination of the alkoxy groups upon heating.^19^ Therefore, the weight loss observed for PHPO_0.21_ is likely attributed to the elimination of alkoxy side groups at the phosphoester crosslinking sites (Fig. 6b). This elimination leads to increasing branch numbers at the crosslinking sites, which alters the mechanical properties.^32^ In contrast, the methyl group substituents associated with the phosphonate crosslinking sites in PHPC_0.21_ are do not participate in bond exchange reactions. Thus, this absence is consistent with the preservation of network structure and mechanical properties.

**Fig. 6:**
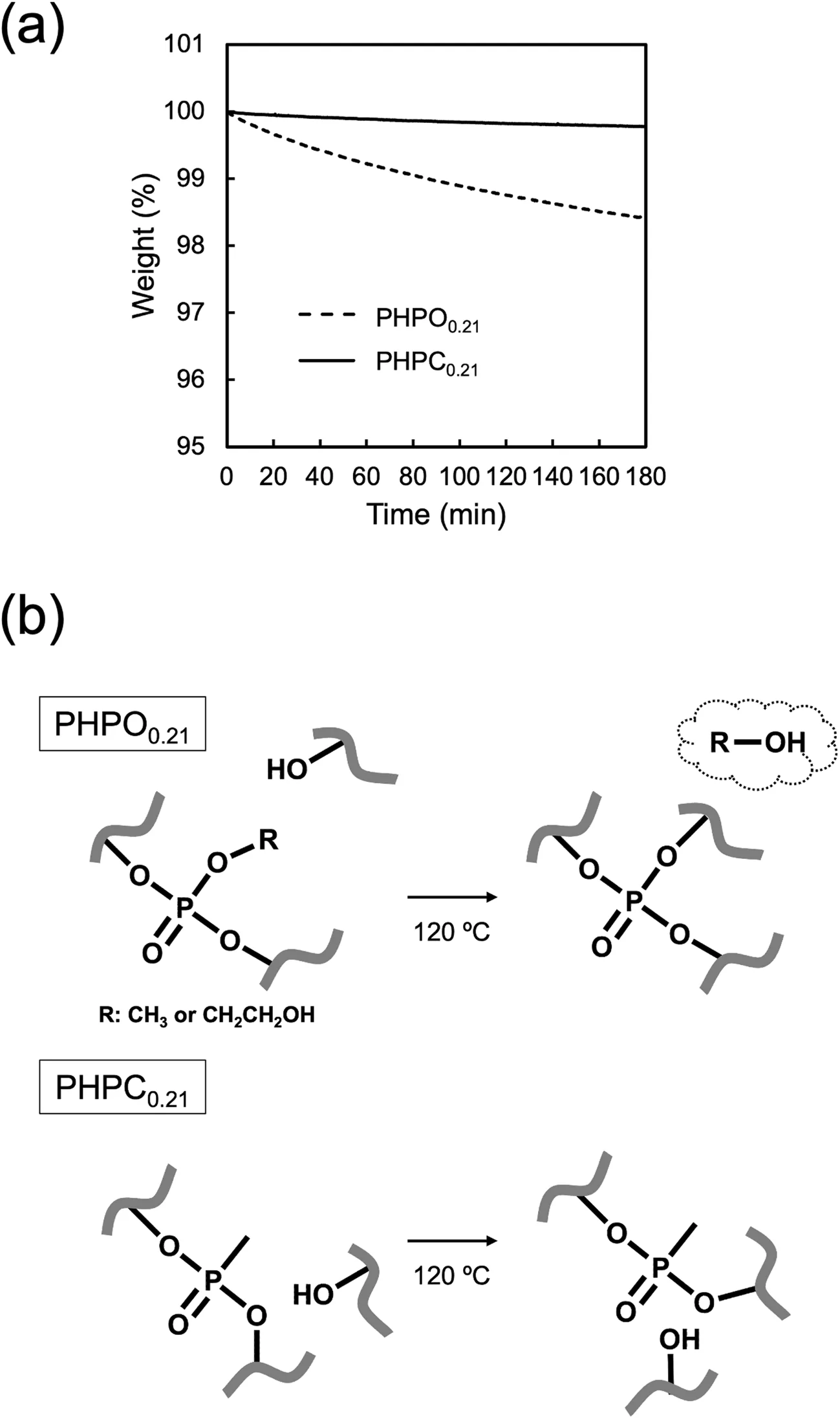
(a) Isothermal TGA curves of PHPO_0.21_ and PHPC_0.21_ measured at 120 °C. (b) Schematic of the proposed structural changes associated with transesterification in PHPO_0.21_ and PHPC_0.21_ during thermal treatment.

The thermal-healing behavior of the networks was subsequently investigated to assess whether bond exchange remained active during heating. No apparent healing was observed for PHEMA or PHPO_0.21_, whereas PHPC_0.21_ exhibited repeatable healing under the applied conditions (Fig. 7). Furthermore, tensile testing of healed PHPC_0.21_ specimens showed that, after 6 h of thermal treatment, ∼99% relative of the tensile toughness of the original sample was recovered (Fig. S3 and Table S2).

**Fig. 7:**
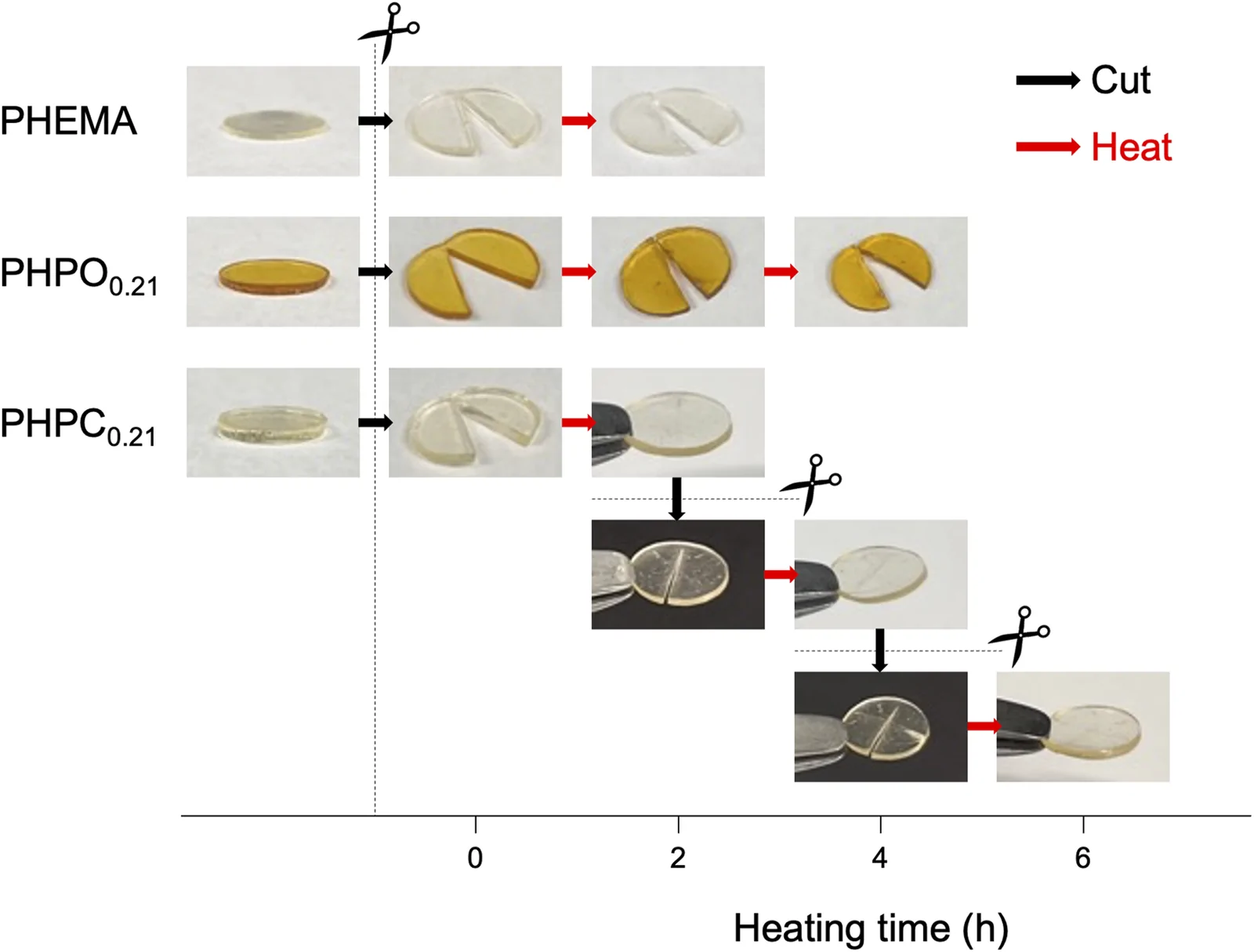
Thermal-healing behavior of PHEMA, PHPO_0.21_, and PHPC_0.21_.

Phosphoester linkages are generally considered to exhibit higher exchange reactivity than phosphonate linkages.^33,34^ Nevertheless, PHPO_0.21_ showed no detectable healing under the present conditions. This behavior is likely associated with irreversible structural changes that occur during thermal curing and subsequent thermal treatment. Preparation of PHPO_0.21_ required curing at elevated temperature for 2 days, which may have promoted alkoxy-group elimination and the formation of more highly connected crosslinking sites. Consistent with this possibility, the Young's modulus of the original PHPO_0.21_ was more than 5.1 times higher than that of PHPC_0.21_ (Fig. 5), whose phosphonate crosslinking motif retains a two-arm structure. Consequently, acquiring reliable stress relaxation profiles for PHPO_0.21_ at elevated temperatures (>160 °C) was technically inaccessible, as its extremely high relaxation modulus exceeded the measurement limit of our rheometer. Nevertheless, in our previous report,^27^ the activation energy (*E*_a_) for the transesterification was determined to be 65.4 kJ mol^−1^ for a film composed of the same polymer matrix. Given that *E*_a_ is theoretically independent of the dynamic bond concentration, this confirms that the present phosphoester DCN inherently possesses dynamic exchangeability. Although it retains this intrinsic potential, previous studies have demonstrated that the exchange dynamics of DCNs depend strongly on the functionality of the dynamic linkage, with an increased number of connecting arms associated with a higher activation energy and a longer stress-relaxation times.^35^ Thus, the formation of more highly connected structures in PHPO_0.21_ may substantially restrict bond exchange and consequently suppress thermal healing. In contrast, the phosphonate crosslinking structure in PHPC_0.21_ remained stable during thermal treatment, as indicated by the absence of detectable mass loss and the retention of its mechanical properties. This structural stability allows the dynamic P–O–C bonds to retain their exchange capability during repeated heating, enabling continuous network rearrangement and healing at the fractured interface. The occurrence of thermally activated bond exchange in PHPC_0.21_ is further supported by stress-relaxation measurements, which yielded an apparent *E*_a_ of 122.0 kJ mol^−1^ (Fig. S2a and b). Collectively, these results demonstrate that the phosphonate-based network provides superior structural stability during thermal activation while retaining sufficient dynamic bond-exchange capability to enable repeatable healing.

### Flame retardancy of phosphoester- and phosphonate-based DCNs

Flame retardancy is commonly imparted to polymers through the incorporation of flame-retardant additives or fillers, which can increase melt viscosity and stiffness and, in some cases, adversely affect processability and mechanical properties.^36,37^ In contrast, the phosphorus-containing crosslinking structures employed in the present networks provide flame-retardant functionality without the addition of flame-retardant additives.

To qualitatively demonstrate this intrinsic functionality, the relative combustion behaviors of the specimens were evaluated under direct flame exposure. Thin-film specimens were utilized for this purpose to enable a clear visual comparison of their self-extinguishing capabilities.

Each specimen was exposed to a flame for 6 s, after which the ignition source was removed. The results are shown in Fig. 8 and SI Videos 1–3. Phosphorus-free PHEMA did not self-extinguish after removal of the ignition source and underwent complete combustion following the flame exposure. In contrast, PHPO_0.21_ and PHPC_0.21_ extinguished after ignition source removal. However, PHPO_0.21_ did not extinguish immediately and continued burning for 10 s after ignition source removal. In contrast, PHPC_0.21_ retained its instantaneous self-extinguishing behavior.

**Fig. 8:**
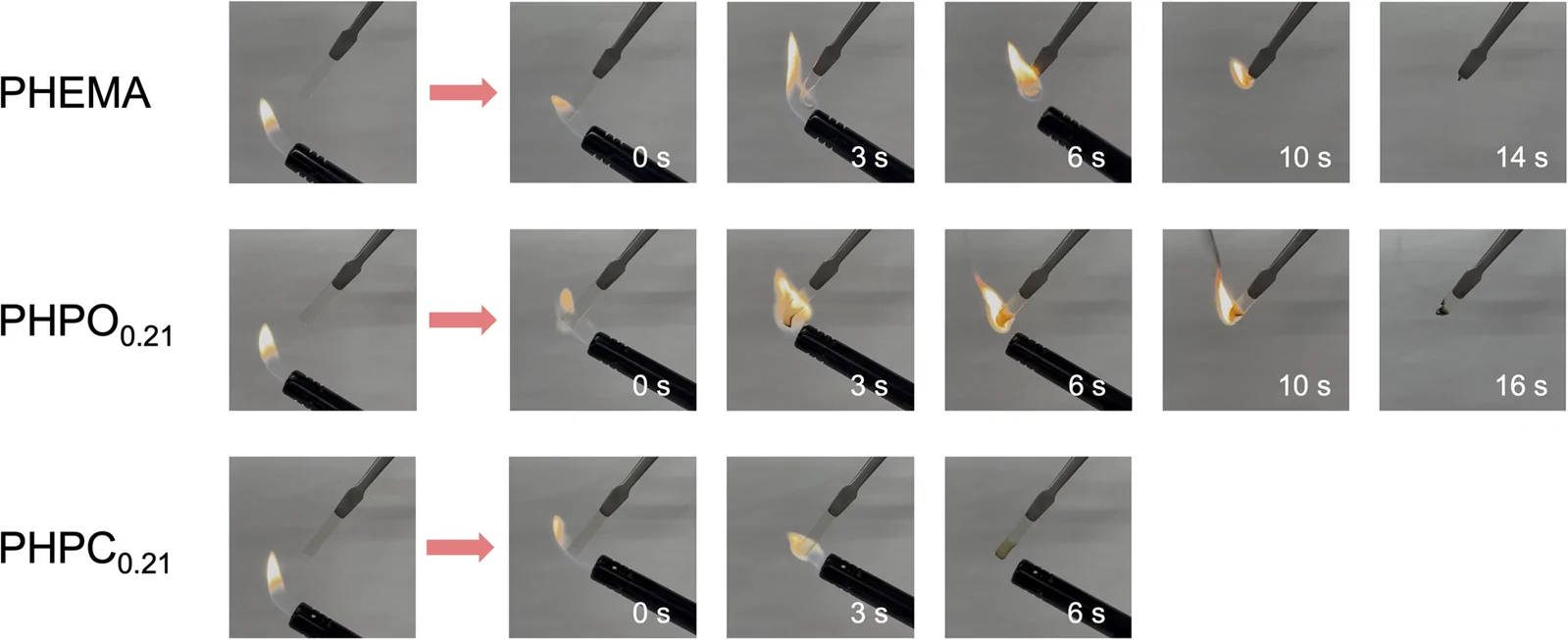
Representative snapshots from combustion-test videos of PHEMA, PHPO_0.21_, and PHPC_0.21_.

After flame exposure, PHPO_0.21_ was covered with a char layer, with PHPC_0.21_ solely restricted to the edges. SEM observations of the surfaces and cross-sections of the residues further revealed distinct internal morphologies (Fig. 9). For PHPO_0.21_, a rough char-derived structure covered the entire surface, and cross-sectional observation confirmed a porous structure not only at the surface layer but also substantially into the interior of the specimen. Conversely, the PHPC_0.21_ surface exhibited numerous cracks, and porous structures were mainly localized to the superficial layer of the flame-exposed edges, with only cracks present in the interior. These differences are attributed to the difference in sustained combustion time. This prolonged combustion time (10 s post-ignition) allowed thermal energy to propagate further into the bulk of PHPO_0.21_, promoting thermal degradation and gas evolution throughout the specimen, generating a continuous porous structure. In contrast, rapid PHPC_0.21_ extinction limited the duration of heat exposure, restricting thermal damage and pore formation exclusively to the regions directly exposed to the flame, keeping internal thermal damages to an absolute minimum.

**Fig. 9:**
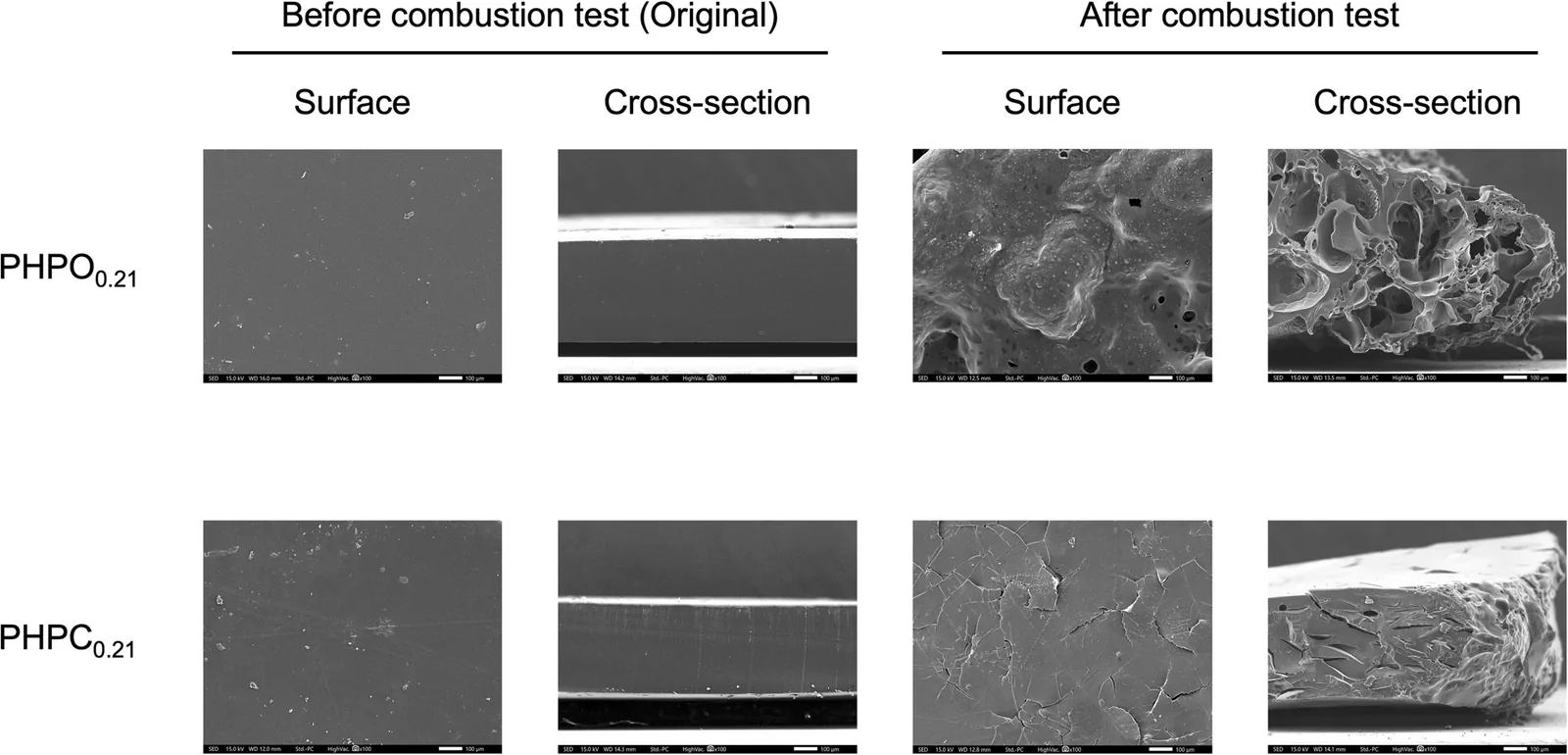
SEM images of the surface and cross-section of PHPO_0.21_ and PHPC_0.21_ before and after the combustion test.

The contrasting combustion behavior can be interpreted in terms of the different flame-retardant mechanisms governed by the phosphoester and phosphonate linkages. Phosphorus-based flame retardants generally suppress combustion through condensed- and gas-phase mechanisms, depending on their chemical structure and decomposition pathways. The preferred pathway is dictated by the specific chemical bonds present in the molecular structure. In phosphoesters, such as triphenyl phosphate^38^ or resorcinol bis(diphenyl phosphate),^39^ the constituent P–O bonds are highly stable and resist thermal radical cleavage. However, they preferentially undergo heterolytic decomposition, such as β-elimination, which generates phosphoric acid, followed by polyphosphoric acid formation through subsequent dehydration condensation. Acting as a strong acid catalyst, this polyphosphoric acid promotes the dehydration and carbonization of the surrounding polymer backbone, forming a robust protective char layer that shields the underlying material from thermal energy and oxygen. This process fundamentally drives the condensed-phase flame-retardant mechanism.^12^ In contrast, phosphonate structures, such as the well-known dimethyl methylphosphonate,^40^ containing P–C bonds may additionally promote gas-phase flame inhibition.^21^ Because the typical bond dissociation energy of a P–C bond (272 kJ mol^−1^) is significantly lower than that of a P–O bond (360 kJ mol^−1^),^41^ P–C bonds are more susceptible to homolytic cleavage than P–O bonds under appropriate thermal conditions.^22^ Consequently, phosphorus-containing radical species may be generated during combustion and released into the gas phase. These radicals have been proposed to inhibit flame propagation by scavenging high-energy radicals involved in the combustion chain reaction. This instantaneous termination of combustion is likely driven by the dominant gas-phase flame-retardant mechanism.^16,42^ Collectively, PHPO_0.21_ and PHPC_0.21_ exhibited effective flame-retardant behavior; however, immediacy of self-extinguishment differed markedly. While direct analysis of the combustion products is required for definitive proof, we hypothesize based on these observed burning behaviors and their chemical structures that PHPO_0.21_ primarily acts through condensed-phase char formation, whereas PHPC_0.21_ may additionally provide gas-phase flame inhibition. This hypothesized rapid gas-phase action could provide an advantage under the severe combustion conditions encountered by thin films, where rapid heat propagation can occur before a protective char barrier is completely established. To quantitatively evaluate the intrinsic combustion properties and heat release behaviors underlying these macroscopic observations, microscale combustion calorimetry (MCC) measurements were subsequently conducted. As shown in Fig. 10 and Table S3, the MCC results may reveal distinct differences in the inherent flame retardancy and mechanistic pathways between PHPO_0.21_ and PHPC_0.21_. PHPC_0.21_ exhibited a significantly lower peak heat release rate (pHRR) of 241.2 W g^−1^ compared to PHPO_0.21_ (368.3 W g^−1^), indicating a substantially reduced fire hazard. Additionally, the fire growth capacity (FGC), an indicator of fire spread risk, was significantly lower for PHPC_0.21_ (175.9 J (g K)^−1^) than for PHPO_0.21_ (225.8 J (g K)^−1^). These differences reflect the distinct flame-retardant mechanisms of the two networks. The yield of pyrolysis residue (*Y*_p_) of PHPO_0.21_ was 9.0%, which is approximately 2.8 times higher than that of PHPC_0.21_ (3.2%). Consequently, the total heat release (THR) of PHPO_0.21_ (18.4 kJ g^−1^) was slightly lower than that of PHPC_0.21_ (18.8 kJ g^−1^). These results suggested that PHPO_0.21_ primarily relies on a condensed-phase mechanism, wherein a robust char layer physically traps combustible fragments and acts as a barrier against heat and oxygen. Conversely, although PHPC_0.21_ exhibited a much lower char yield (3.2%)—indicating that most of its mass volatilized—its pHRR was substantially suppressed. While a larger volume of volatilized compounds typically leads to a higher HRR,^43^ this contradictory behavior suggests that the gases released from PHPC_0.21_ are inherently less combustible. Indeed, the specific heat of combustion of the fuel gases (*h*_c,gas_) for PHPC_0.21_ (19.5 kJ g^−1^) was lower than that for PHPO_0.21_ (20.2 kJ g^−1^). This observation strongly supports our hypothesis that PHPC_0.21_ provides additional gas-phase flame inhibition by releasing volatile phosphorus-containing radical scavengers during thermal decomposition. In conclusion, the rapid flame extinction of PHPC_0.21_ observed in the macroscopic tests can be attributed to this effective gas-phase action, which compensates for its lower char-forming ability.

**Fig. 10:**
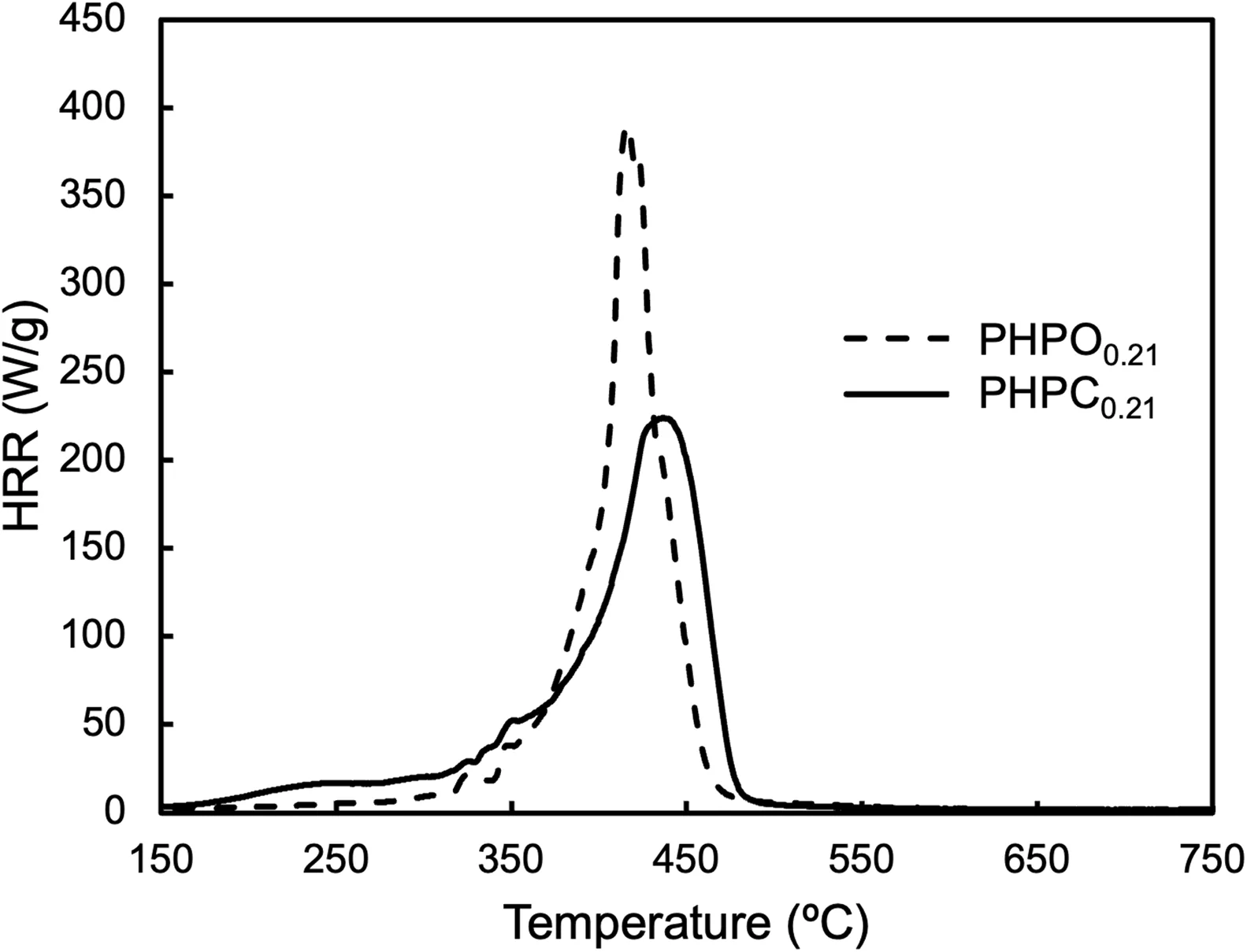
Heat release rate (HRR) plots of PHPO_0.21_ and PHPC_0.21_ obtained from microscale combustion calorimetry (MCC) measurements.

## Conclusions

In this study, phosphoester- and phosphonate-based DCNs were prepared by crosslinking PHEMA with cyclic phosphorus compounds. The incorporation efficiencies of phosphoester and phosphonate units into the films was different due to the distinct ring-opening reactivities of the corresponding cyclic phosphorus compounds. Furthermore, the changes in the mechanical properties of the films with identical phosphoester or phosphonate units incorporation ratios were compared to induce bond exchange reactions. The phosphoester-based DCNs underwent irreversible structural changes during thermal treatment, accompanied by mass loss and an increase in Young's modulus. These changes were likely attributed to the elimination of alkoxy-group substituents at the phosphoester-crosslinking sites, which altered the network architecture and diminished its ability to undergo dynamic bond exchange and thermal healing. Conversely, the phosphonate-based DCNs preserved their mechanical properties during thermal treatment and exhibited repeatable healing, highlighting their structural stability under repeated thermal activation. Furthermore, the two networks exhibited distinct flame-retardant behaviors. Both networks showed self-extinguishing behavior after flame exposure; however, they exhibited a marked difference in the immediacy of flame extinction. The phosphoester-based DCNs formed an extensive char structure but required a longer time to extinguish, resulting in substantial internal thermal damage. In contrast, the phosphonate-based DCNs retained rapid self-extinguishing behavior and limited thermal damage primarily to the flame-exposed regions. Furthermore, MCC measurements quantitatively corroborated these macroscopic burning behaviours from the perspective of heat release. The phosphonate-based DCNs showed an effectively suppressed heat release rate, and this reduction in heat generation directly contributed to its quick flame quenching and rapid self-extinguishment. On the other hand, the phosphoester-based DCNs exhibited sustained heat release during combustion, and it is considered that this prolonged exposure to generated heat ultimately led to the observed deep internal thermal damage.

Therefore, a comparison of the properties of phosphoester and phosphonate crosslinkages demonstrated that incorporating phosphonate groups into the DCNs preserves stable dynamic properties and mechanical integrity while endowing the material with flame-retardant properties. These findings help in the development of next-generation multifunctional DCNs.

## Author contributions

Kazuho Maeda: methodology, formal analysis, investigation, funding acquisition, writing – original draft, writing – review & editing. Yota Okuno: methodology, formal analysis. Yasuhiko Iwasaki: conceptualization, formal analysis, funding acquisition, resources, supervision, writing – original draft, writing – review & editing.

## Conflicts of interest

There are no conflicts to declare.

## Supplementary Material

RA-016-D6RA08494J-s001

RA-016-D6RA08494J-s002

RA-016-D6RA08494J-s003

RA-016-D6RA08494J-s004

## Data Availability

The data supporting this article have been included as part of the supplementary information (SI). Supplementary information is available. See DOI: https://doi.org/10.1039/d6ra08494j.
