# Supplementary material for: Fabrication of healable and flame-retardant polymer films based on phosphonate dynamic covalent crosslinking networks
Source: RSC Adv. 2026 Oct 21;16(48):65134–42. doi: 10.1039/d6ra08494j (PMC13685702; doi:10.1039/d6ra08494j)
Supplement: Supplementary file 1 [file RA-016-D6RA08494J-s004.pdf]

Supplementary Information (SI) for RSC Advances.
This journal is © The Royal Society of Chemistry

Supplementary Information

Fabrication of healable and flame-retardant polymer films based on phosphonate dynamic covalent crosslinking networks

Kazuho Maeda[1]，Yota Okuno[2,3]，Yasuhiko Iwasaki[2,3]*

[1] Graduate School of Science and Engineering, Kansai University, 3-3-35, Yamate-cho, Suita, Osaka 564-8680, Japan

[2] Department of Chemistry and Materials Engineering, Faculty of Chemistry, Materials and Bioengineering, Kansai University, 3-3-35 Yamate-cho, Suita-shi, Osaka 564-8680, Japan

yasu.bnt@kansai-u.ac.jp

[3] ORDIST, Kansai University, 3-3-35 Yamate-cho, Suita-shi, Osaka 564-8680, Japan

* *Corresponding author*

METHODS

Rheological measurements

Rheological characterizations, including amplitude sweeps and stress relaxation experiments, were conducted on an Anton Paar MCR102 rheometer (Austria). Isothermal stress relaxation profiles, represented by the relaxation modulus (G(t)), were recorded at 5 temperature points ranging from 160 to 200 °C under a fixed shear strain of 1%. For an accurate comparison of the relaxation kinetics, all G(t) data were normalized with respect to the modulus value at a reference time of 10 s. These normalized curves were subsequently fitted to a stretched exponential model (also normalized to 10 s) to extract the characteristic relaxation times (τ*). An Arrhenius relationship was then plotted using these τ* values, and the apparent activation energy (E_a) of the bond exchange process was calculated from the linear slope of the plot, as defined by Equation (1):

$$\ln \square(t) = \ln \square_0 + \frac{E_a}{RT} \quad (1)$$

RESULTS

Table S1 Summary of the mechanical properties of PHEMA, $PHPO_{0.21}$ and $PHPC_{0.21}$. The Young's modulus was determined from the slope of the initial linear region of the stress–strain curves. All data are presented as the mean ± standard deviation derived from at least three independent measurements (n=3).

Sample	Young's modulus (MPa)	Tensile strength (MPa)	Elongation at break (%)
PHEMA	1553.3±245.0	43.0±2.2	4.3±1.2
$PHPO_{0.21}$	479.6±59.3	18.1±3.3	59.2±9.0
$PHPC_{0.21}$	94.6±39.2	3.0±0.9	284.8±11.5

Table S2 Tensile toughness of the original and thermally healed $PHPC_{0.21}$. The toughness values were calculated from the integrated area under the corresponding stress–strain curves. Data are presented as the mean ± standard deviation derived from at least three independent measurements (n=3).

Sample condition	Tensile toughness ($MJ \cdot m^{-3}$)
Original $PHPC_{0.21}$	5.4±1.6
$PHPC_{0.21}$ after healing by heating for 6 hours	5.5±1.6

Table S3 MCC-derived parameters of $PHPO_{0.21}$ and $PHPC_{0.21}$, namely: peak heat-release rate (pHRR), total heat release (THR), specific heat of combustion of fuel gases ($h_{c,gas}$), yield of pyrolysis residue (Y_p) and the flame growth capacity (FGC).

Sample	pHRR ($W \cdot g^{-1}$)	THR ($kJ \cdot g^{-1}$)	$h_{c,gas}$ ($kJ \cdot g^{-1}$)	Y_p (%)	FGC ($J \cdot (g \cdot K)^{-1}$)
$PHPO_{0.21}$	368.3±14.0	18.4±0.3	20.2±0.1	9.0±1.0	225.8±7.7
$PHPC_{0.21}$	241.2±16.2	18.8±0.4	19.5±0.4	3.2±0.1	175.9±5.6

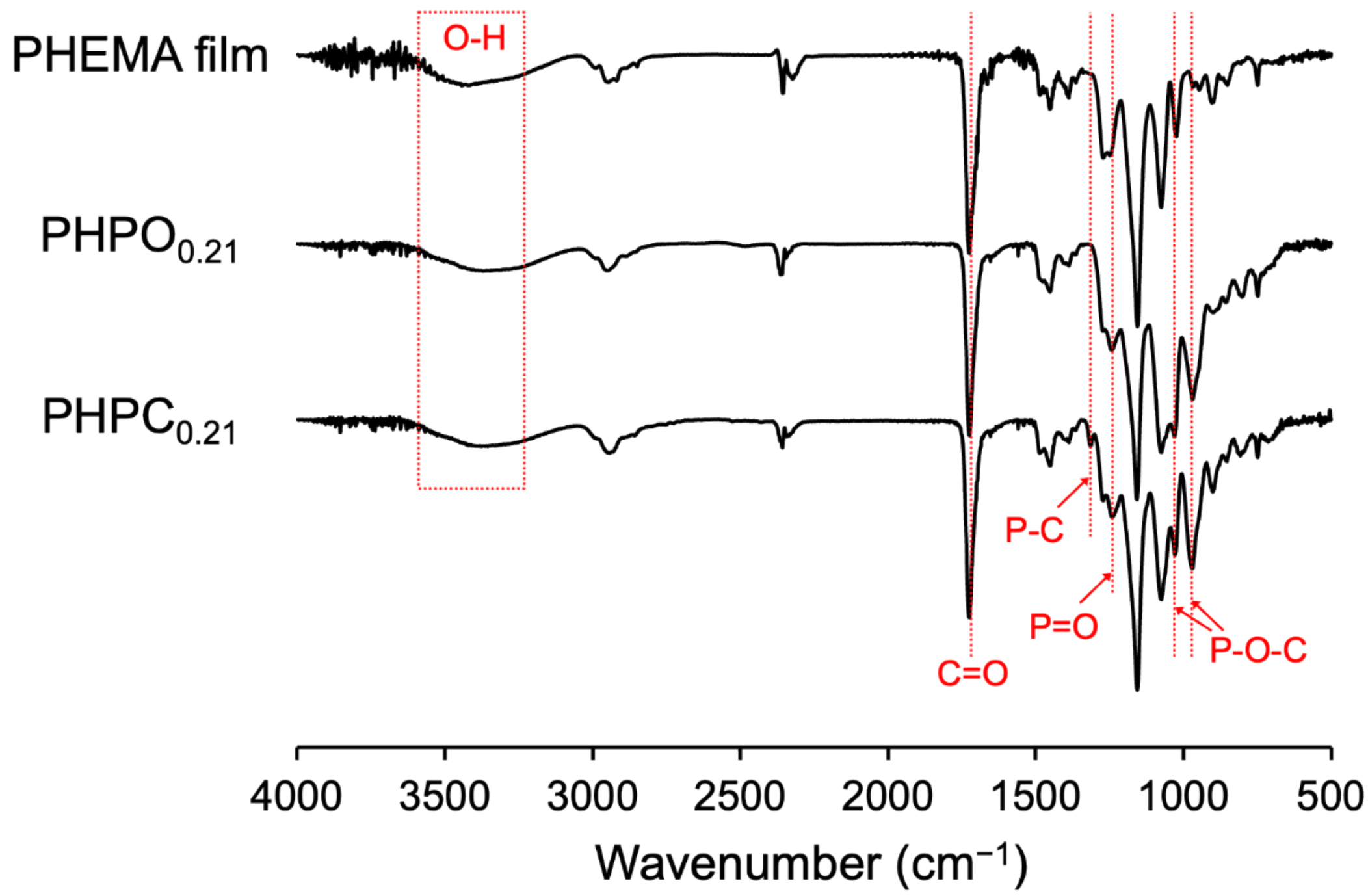

Fig. S1 Full ATR-FTIR spectra of PHEMA, $PHPO_{0.21}$, and $PHPC_{0.21}$.

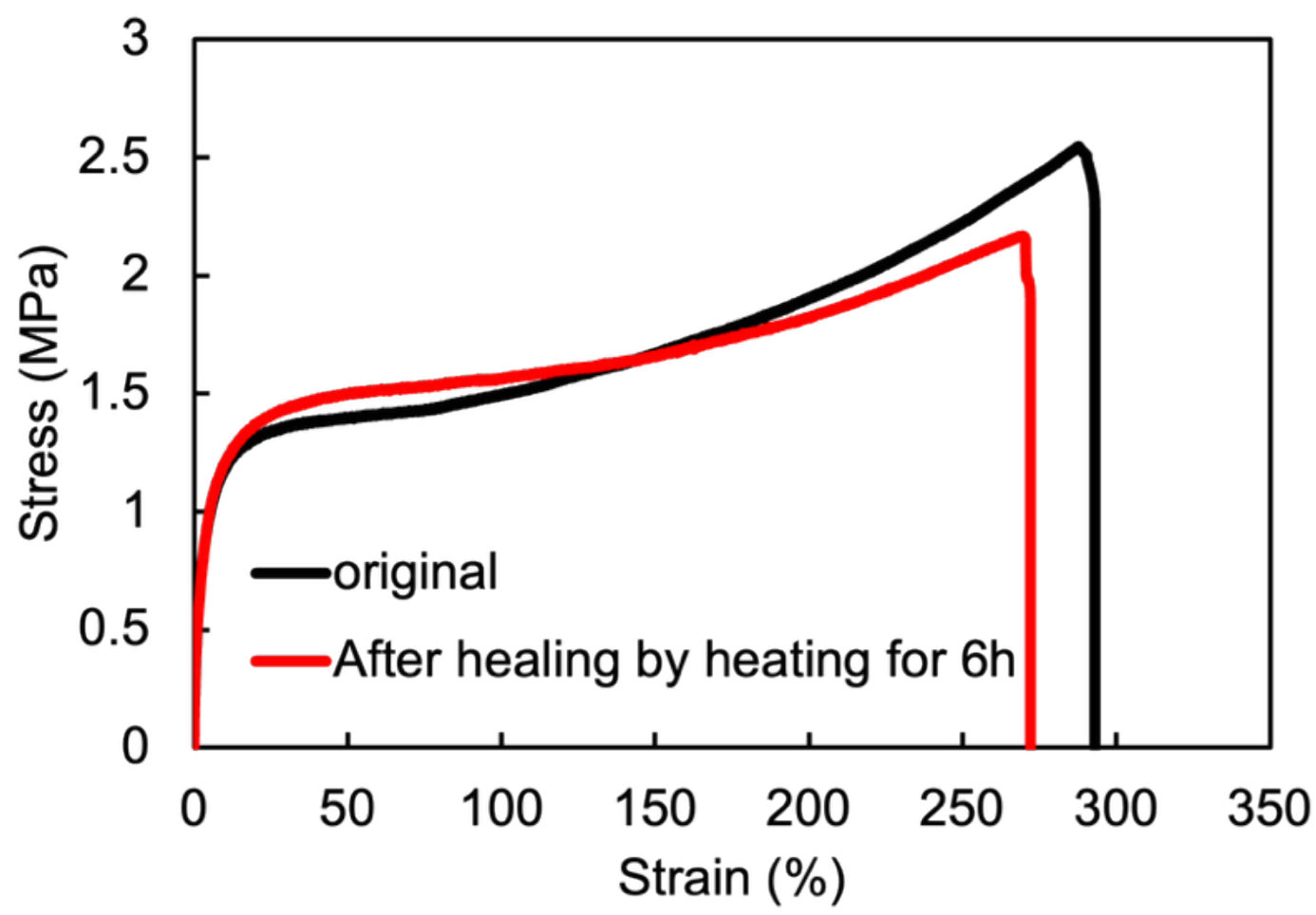

Fig. S2 Stress–strain curves of the original and thermally healed $PHPC_{0.21}$. The tensile tests were performed using dumbbell-shaped specimens at a constant crosshead speed of 5.0 $\mathrm{mm \cdot min^{-1}}$ at room temperature. The thermally healed sample was prepared by bringing the fractured surfaces into intimate contact under an applied compressive load at 120 °C for 6 h. The curves shown are representative of at least three independent measurements.

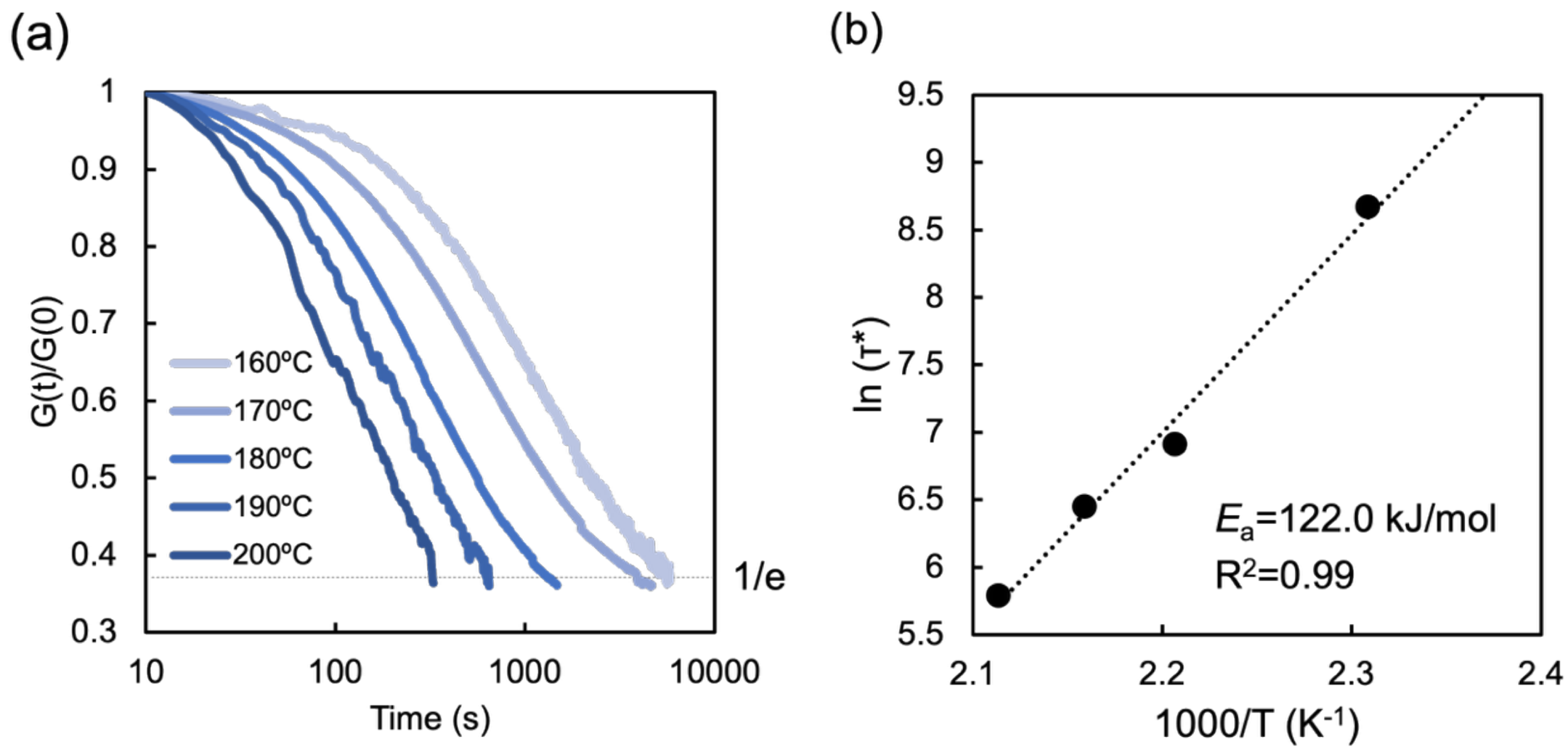

Fig. S3 (a) Stress-relaxation curves measured at various temperatures ranging from 160 to 200 °C with a constant strain of 1%. The characteristic relaxation times (τ^*) were extracted by linear fitting of the relaxation data. The stress values were normalized to the initial stress. (b) Arrhenius plot of the relaxation times for $PHPC_{0.21}$. The apparent activation energy (E_a) was calculated to be 122.0 kJ mol^{-1} from the slope of the linear regression ($R^2 = 0.99$).
